# Semantics and syntax effects on event-related fields during speech comprehension: a MEG study

**DOI:** 10.3389/fnhum.2026.1759177

**Published:** 2026-04-02

**Authors:** Natalia Zhozhikashvili, Olga V. Sysoeva, Elizaveta Makarova, Olga V. Martynova

**Affiliations:** 1 Laboratory for Cognitive Research, HSE University, Moscow, Russia; 2 Laboratory of Human Higher Nervous Activity, Institute of Higher Nervous Activity and Neurophysiology, Russian Academy of Science, Moscow, Russia; 3 Sirius Center for Cognitive Sciences, Sirius University of Science and Technology, Sirius, Russia; 4 Faculty of Biology and Biotechnology, Joint Department with RAS Shemyakin-Ovchinnikov Institute of Bioorganic Chemistry, HSE University, Moscow, Russia; 5 Centre for Cognition and Decision Making, Institute for Cognitive Neuroscience, HSE University, Moscow, Russia

**Keywords:** auditory perception, event-related fields, MEG, natural speech comprehension, semantic dissimilarity, word class

## Abstract

**Introduction:**

Understanding how the brain processes language during natural speech comprehension remains a key challenge. This study tested the feasibility of using an event-related field (ERF) approach to examine the spatiotemporal distribution of brain activity evoked during continuous speech comprehension.

**Methods:**

We used MEG and cluster-based permutation analysis of ERFs to examine how semantic dissimilarity and word class modulate neural responses during continuous speech comprehension.

**Results:**

Words with high semantic dissimilarity elicited a late ERF effect at left temporal sensors (616-666 ms). This effect remained robust after controlling for word duration and subsequent words. Function words elicited an early ERF (166–312 ms) at left parieto-temporal sensors and a later effect (470–654 ms) at temporal sensors.

**Discussion:**

The late effect for words with high semantic dissimilarity may be linked to P600-like activity or other late semantic integration processes. The early and late effects for function words may reflect syntactic processing and the prediction of upcoming words. Together, these findings suggest that semantic dissimilarity and word class differentially shape the temporal dynamics of ERFs during natural speech comprehension.

## 1 Introduction

The processing of language involves complex neural mechanisms that allow the brain to interpret and integrate both semantic and syntactic information. One key factor that can influence semantic processing is *semantic dissimilarity*, or the degree to which a word’s meaning differs from the semantic context established by the preceding words (Broderick et al., 2018; Frank and Willems, 2017). In natural speech comprehension, semantic dissimilarity is particularly important as it can increase the cognitive load required to integrate a word into its surrounding discourse. Understanding how the brain handles semantically distinct words can provide valuable insights into the neural mechanisms underlying language comprehension in real-world, conversational contexts. Importantly, semantic dissimilarity is not the same as word expectancy or probability, which have been more extensively described in the literature. For example, in the sentence “The boy will eat cake,” the word “cake” is expected or predictable, even though it is semantically distinct from the other words in the sentence, whereas “cake” and “pie” are semantically similar (Frank and Willems, 2017).

One of the most conventional methods for studying brain activity during speech perception is the analysis of event-related potentials (ERPs; Kaan, 2007, Molfese et al., 2008, Harwood et al., 2017, Rebreikina et al., 2021, Larionova and Martynova, 2022, Liaukovich et al., 2022; Neklyudova et al., 2025). These electrical brain responses, evoked by isolated linguistic stimuli, such as phonemes, words or sentences, provide valuable insights into the mechanisms underlying the perception and understanding of speech. The N400 and P600 are among the most extensively studied ERP components in language research. The N400, peaking between 300 and 500 ms after word onset, was initially linked to semantically incongruent words (Kutas and Hillyard, 1980) and has been shown to be more pronounced for meaningless pseudowords and infrequent words (Kutas and Federmeier, 2000; Van Petten and Kutas, 1990). The P600, in contrast, is classically associated with the processing of syntactic anomalies, but it can also be elicited by certain types of semantic violations, particularly when they conflict with sentence structure (Gouvea et al., 2010; Osterhout and Holcomb, 1992; van Herten et al., 2005; Kuperberg, 2007). The Retrieval-Integration Theory proposes that the N400 reflects lexical retrieval—the access of a word’s meaning from memory—while the P600 reflects integration of that meaning into the evolving sentence representation (Brouwer et al., 2017), with greater amplitudes indicating increased integration difficulty (Aurnhammer et al., 2021).

ERPs associated with word processing have traditionally been examined in the context of unnatural speech streams that include semantic or syntactic errors (Service et al., 2007; Hagoort and Brown, 2000; Kutas and Federmeier, 2011; Friederici et al., 1993), and a methodological shift towards more naturalistic conditions in speech perception experiments has only emerged in recent years (Alday, 2019). Recent studies have demonstrated that the brain tracks the semantic content of natural speech in a time-locked manner, with neural responses being sensitive to the meaning carried by individual words in narrative context (Broderick et al., 2018; Rogachev and Sysoeva, 2024; Soghoyan et al., 2025). This phenomenon, known as neural tracking, involves cortical activity that reflects the brain’s processing of acoustic and linguistic features of words. Neural tracking is commonly examined using convolution methods, most notably temporal response functions (TRFs) or coherence analysis (Broderick et al., 2018; Gillis et al., 2021). In particular, for the speech listening condition, the TRF method uses different predictors for building a model of convolution of continuous brain activity with auditory speech envelope or discrete events such as word onsets as well as word semantic dissimilarity (Broderick et al., 2022; Mesik et al., 2021; Zhou et al., 2022; Ovakimian et al., 2025). This approach has shown that the brain responses to semantically complex words are reflected in a prominent negativity, around 380–600 ms after word onset, which was interpreted as N400 by the authors (Broderick et al., 2018).

We asked whether a modified ERP approach could provide an additional understanding of brain responses to natural speech listening. The modification involved averaging brain activity associated not with the same linguistic events (like semantically incongruent words), but with semantically similar versus contextually distinct words, using a semantic dissimilarity metric, which has been shown to be an effective predictor of brain activity in convolutional methods for tracking of natural speech processing, such as TRF. The analogous event-related approach was invented in the reading word-by-word condition for the streaming speech processing by Neklyudova et al. (2025). In this study, we analyzed magnetoencephalography (MEG) event-related fields (ERFs) to compare semantically close and distant content words in continuous narrative Russian speech, in order to examine how semantic dissimilarity modulates brain responses during natural speech comprehension. The ERP approach has some methodological limitations compared to TRF and other regression-based analyses. It requires dividing stimuli into categorical groups (e.g., words with high dissimilarity vs. words with low dissimilarity), which introduces somewhat arbitrary thresholds and discards information about intermediate values, thereby reducing statistical power and making it difficult to capture complex, non-monotonic (e.g., U-shaped) dependencies. At the same time, the ERP approach is computationally less demanding, requiring substantially less memory than TRF, which makes it feasible to analyze a much larger number of words with long epochs without access to high-performance computing resources. Moreover, contrasting extreme conditions may increase sensitivity to non-linear but monotonic effects that primarily emerge at the edges of the distribution — effects that could be smoothed out or entirely missed by the linear regression approach used by Broderick et al. (2018). Finally, a categorical ERP approach makes it possible to interpret the results in terms of well-known ERP components, facilitating comparison of convolution/regression methodological findings with conventional neurolinguistic knowledge.

Thus, we aimed to compare results of the ERFs locked to semantically similar versus semantically distinct words with the described findings of Broderick et al. (2018). Similar patterns (the ERF between 380 and 600 ms) might be anticipated, and agreement between the two methods would provide evidence for the robustness and validity of both. Thus, semantic dissimilarity is thought to increase the cognitive load required for integrating a word into its surrounding discourse, potentially leading to distinct neural responses, particularly in the late stages of processing (Broderick et al., 2018). However, due to the described methodological differences and language differences, divergences in peak latencies or effect magnitudes are plausible. The morphological complexity of Russian (including suffixes and inflectional endings), along with different word durations and flexible word order, which complicates syntactic parsing, may lead to delayed ERP peaks due to increased processing demands. For example, in a Russian-language study (Ovakimian et al., 2025), the semantic-dissimilarity regressor predicted an earlier TRF response (200–400 ms), which the authors interpreted as evidence for early bilateral semantic processing. However, due to the computational demands of the TRF method, the study used few words and short windows around word onsets, which could affect generalizability.

In addition to investigating the role of semantic dissimilarity, this study also compares function and content words to explore how word type influences ERF responses during the auditory processing of natural speech. In language, two main word classes are distinguished: (1) open-class words, or content words, which include nouns, verbs, adjectives, adverbs, and interjections; and (2) closed-class words, or function words, which include pronouns, numerals, conjunctions, particles, prepositions, and auxiliary verbs. Function words carry little to no lexical-semantic content, their meaning in isolation is minimal or even nonexistent. However, they play a crucial syntactic role, shaping sentence structure and indicating relationships between words (e.g., case or type of dependency). Prior EEG studies provide classical evidence for differences between these word classes: Pulvermüller et al. (1995) reported left-lateralized N150 and N260 ERP components for function words, reflecting early engagement of perisylvian regions in grammatical processing. Function words, as well as content words, can also elicit N400 effects associated with the anticipation of upcoming words or their grammatical features (DeLong et al., 2005). Münte et al. (2001) found that content words evoked stronger N400 responses, which they interpreted as reflecting the greater semantic load and contextual integration required by content words. When analyzing continuous speech, Gillis et al. (2021) compared TRF responses to content and function words and found no significant differences, suggesting that neural responses to natural speech do not primarily reflect a word’s grammatical class. In contrast, Schilling et al. (2021) analyzed MEG ERF responses to continuous speech, found that content words elicited stronger early (0–250 ms) and late (500–750 ms) responses than function words, and interpreted this as reflecting the higher semantic load and deeper contextual integration required by content words, which may suggest that ERFs may better capture such semantic differences. However, to our knowledge no study of natural speech has yet performed a rigorous group-level statistical analysis of MEG ERFs related to function and content word classes. In the present study, we addressed this gap by applying spatio-temporal cluster-based tests across participants.

In summary, we hypothesized that ERFs to natural speech would be sensitive to semantic load when comparing content and function words, and to semantic dissimilarity when contrasting high- and low-dissimilarity content words. Observing an N400 in these contrasts would support previous findings and support the suitability of MEG ERFs for studying semantic processing in natural speech, while deviations, such as differently distributed effects, could reflect language-specific processing characteristics of Russian or differences between ERF and TRF methodologies.

## 2 Methods

### 2.1 Participants

Twenty seven healthy native-language speaking right-handed adult volunteers participated in the study. The sample size was selected in accordance with the median number of participants in previous studies using MEG to study speech processing (i. e., ~26; Gwilliams et al., 2023; Liu et al., 2020; Houde et al., 2002; Zhao and Kuhl, 2022; Pulvermüller and Shtyrov, 2009). Participants were recruited via announcements in social media. All participants were paid for participation, some of whom were university students who also received course credits for their participation. All participants were native language speakers; they had normal or corrected-to-normal vision; they reported no history of neurological, or mental disorders. The experiments were approved by the Institute of Higher Nervous Activity and Neurophysiology’s ethics committee (№ 0304052023, May 4th, 2023), followed the Helsinki Declaration’s ethical guidelines for human research, and signed informed consent was obtained from all participants.

### 2.2 Auditory speech processing task

During the MEG recording, participants were presented with a short audio-text read in a female voice (and processed by a professional sound engineer) through headphones. The text was a short pop-science article about hedgehogs. The text had a length of 672 words, of which 166 were function (prepositions, conjunctions) and 506 were content words. Text of the story and translated to English text can be viewed in the Supplementary material. While listening, participants were shown a fixation cross on a black background on the screen. After the audio story, the experimenter asked questions about the content of the story in order to track the participant’s involvement. The duration of the story was 4 min and 52 s. Function words (for ex., ‘in’, ‘when’, ‘to’, ‘not’, ‘but’) had mean duration 167 (ms ± 135 ms – standard deviation). Content words (for ex., ‘hedgehogs’, ‘live’, ‘light’) had mean duration 394 ms (±158 ms).

### 2.3 Semantic dissimilarity

To analyze the MEG response to close- and distinct-to-context words, a semantic dissimilarity value was calculated for each word. First, word embeddings were generated using the pre-trained fastText model (cc.ru.300.bin; Grave et al., 2018). The model was trained on a large corpus of Russian text and provides 300-dimensional vector representations for words. Then, semantic dissimilarity of each word was calculated using their vector representations. We compared each word’s vector with the mean vector of all previous words in the sentence, using Pearson correlation as a measure of similarity. The dissimilarity was computed as 1 - Pearson correlation, where higher values indicate greater dissimilarity. The resulting dissimilarity scores reflected how semantically distant each word is from its context (Broderick et al., 2018). The value assigned to the initial word of the text (“hedgehogs”) was set to 1. The vector of the first word in a sentence was compared to the average of all word vectors from the preceding sentence.

Then, the content words were divided into quartiles by semantic dissimilarity values. Groups of words for comparison were identified: ‘close-to-context’ (lower quartile) and ‘distinct-to-context’ (upper quartile). Thus, the MEG response to 127 close-to-context and 127 distinct-to-context content words was analyzed in total. Close-to-context words (for ex., ‘grass’, ‘hedgehogs’, ‘they’, ‘small’, ‘now’) had mean duration 324 ms (±143 ms). Distinct-to-context words (for ex., ‘relatives’, ‘white-bellied’, ‘farm’, ‘meter’) had mean duration 453 ms (±162 ms).

We also analyzed the brain activity elicited by words following the close- and distinct-to-context words to examine whether the late ERF differences we observed were related to the onset of the next word. Before conducting the analysis, we checked that the proportions of function, content, close- and distinct-to-context words were statistically comparable across the analyzed groups using the chi-square test, to ensure that any observed neural differences could not be attributed to uneven distributions of word types, which may differentially engage processing mechanisms.

The word lists can also be seen in the Supplementary material. To obtain word onset and offset markers, the audio story recording was aligned with its textual transcript and manually annotated using the ‘Audacity’ software for spectrogram inspection.

### 2.4 MEG data acquisition

MEG was recorded using a 306-channel detector array (Vectorview; Neuromag, Helsinki, Finland). The data was recorded with a band-pass filter of 0.03–330 Hz, digitized at 1000 Hz. The subjects’ head positions were continuously monitored during MEG recordings. The study was conducted at the unique research facility — “Center for Neurocognitive Research” of the Moscow State University of Psychology and Education.

### 2.5 MEG data preprocessing

The data was first denoised using the Temporal Signal-Space Separation (tSSS) method and adjusted to the expected common head position using MaxFilter™ (v2.2) software. For further pre-processing, we used the MNE-python toolbox (v.0.24.1) as well as custom Python and Matlab scripts. The denoised data was filtered between 0.2 and 40 Hz. Independent component analysis (ICA) was used for correction of biological artifacts. The data was segmented from −0.05 to 1 s relative to the stimulus onset. Such length of epochs was chosen because in natural Russian speech stimuli (words) have a duration of about 0.15–0.60 s (Stepanova, 2011). Then epochs with amplitude exceeding or subceeding 8 standard deviations relative to the mean for at least one channel were rejected. Then a common peak-to-peak signal amplitude threshold 6e-12 T was used to reject epochs (Leiken et al., 2014; Grabot et al., 2025; Cui et al., 2022). At the end, the average number of analyzed epochs was 164.0, 499.7, 125.7, 125.6, for ‘function all’, ‘content all, ‘close-to-context content’, ‘distinct-to-context content’ conditions, respectively.

### 2.6 Event-related field analysis of the MEG data at the sensor level

Magnetometers were used for the sensor-level analysis, as after tSSS they provide equivalent information to gradiometers or combined sensors (Garcés et al., 2017). ERFs to word onset were calculated by averaging baseline-corrected (−0.05–0 s) signals across epochs. For statistical analysis, we compared conditions (close- vs. distinct-to-context words, function vs. content words) to assess whether differences in semantic dissimilarity or word type are reflected in neural responses. To identify significant spatio-temporal clusters of ERFs, we used a permutational cluster-based analysis (function spatio_temporal_cluster_test) with a cluster-forming threshold of *α* = 0.001 and 1,000 permutations (Clarke et al., 2024; Orpella et al., 2022; Lim et al., 2021). Identifying clusters of sensors with similar signals reduces the multiple comparison problem. To compute the actual test statistic, we first calculated the summed *F*-value for each cluster, yielding a single test statistic per cluster. Then a distribution from the data was generated by shuffling the conditions between the samples and recomputing the clusters and the test statistics. Significance of a given cluster was tested by computing the probability of observing a cluster of that size. The cluster significance threshold was set at 0.005. For ERFs visualizations, we used the plot_joint() function (MNE), with topography latencies determined by the peak of the absolute amplitude across the averaged channels for each condition.

## 3 Results

### 3.1 Event-related fields for content close-to-context and content distinct-to-context words

**Figure 1:**
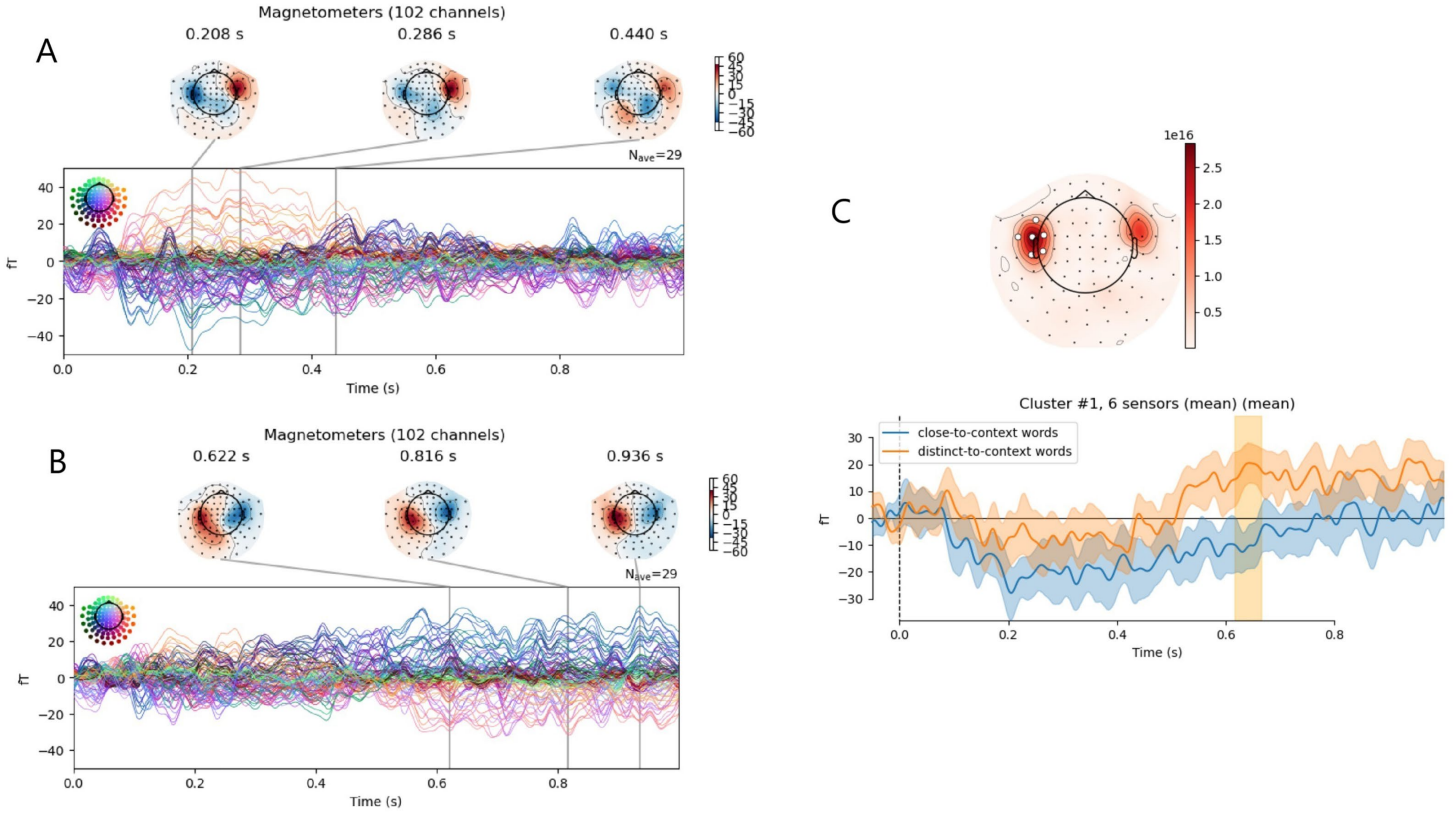
Event-related fields (ERFs) for close- and distinct-to-context words. **(A,B)** ERFs shown for all channels in one timeline with highlighted peaks and their topography: **(A)** close-to-context words, **(B)** distinct-to-context words. **(C)** comparison of ERFs to close- and distinct-to-context words: top panel—topography map with a significant sensor cluster marked in white, bottom panel—ERF averaged across clustered channels; significant time periods are highlighted with yellow color. Confidence interval across participant data is shown.

ERFs related to content words demonstrated peaks at the temporal sensors (see Figures 1A,B). Permutational cluster analysis revealed one significant cluster (Figure 1C). At the left temporal sensors, distinct-to-context words induced more positive ERF from 616 to 666 ms from the word onset, compared to close-to-context words (*F* = 16.7, *p* = 0.002).

To account for potential confounds related to ongoing word perception, we additionally verified the effects in a subset of shorter words (<500 ms), and the results remained unchanged (see Supplementary Figure S1).

Although statistical differences were observed for late time periods, a visual inspection of the ERFs across individual channels revealed the early components of the ERFs at the left lateral parietal sensors (for the figure and its discussion see Supplementary Figure S2).

### 3.2 Event-related fields for function and content words

**Figure 2:**
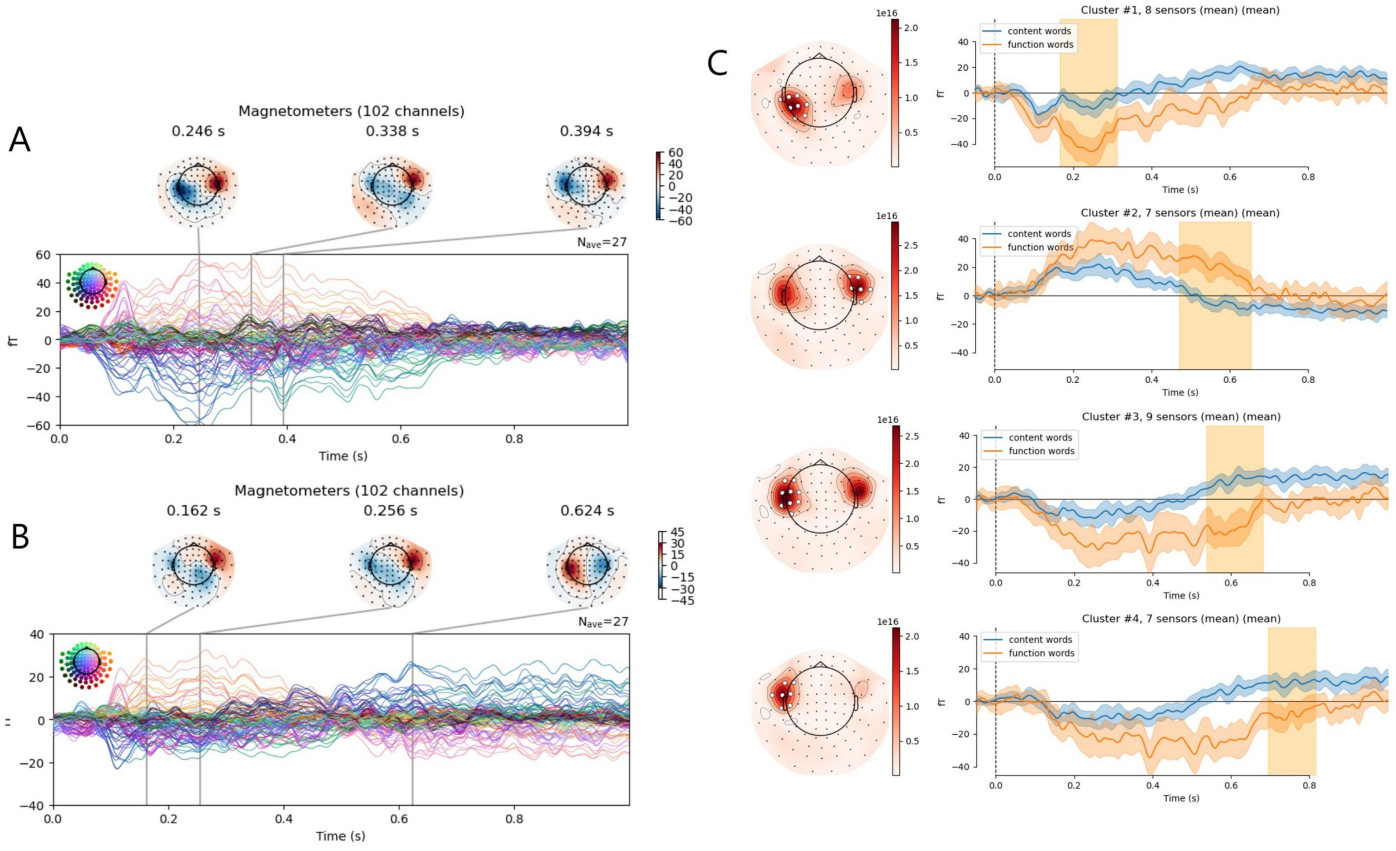
Event-related fields (ERFs) for function and content words. **(A,B)** ERFs shown for all channels in one timeline with highlighted peaks and their topography: **(A)** function words, **(B)** content words. **(C)** comparison of ERFs to function and content words: left panels—topography map with significant sensor clusters marked in white, right panels—ERFs averaged the significant clusters; significant time periods are highlighted with yellow color. Confidence interval across participant data is shown.

ERFs related to analyzed words demonstrated significant peaks with temporal topography (see Figures 2A,B). Permutational cluster analysis revealed four significant clusters that differentiate content and function words. At the left temporal sensors, function words induced more pronounced negative ERF from 166 to 312 ms from word onset (*F* = 13.0 *p* = 0.001; see Figure 2C). At the right temporal sensors, function words induced more pronounced positive ERF from 470 to 654 ms from the word onset (*F* = 15.7, *p* = 0.001; see Figure 2C). Also, at the left temporal sensors, two late significant clusters of the ERF differences were observed: from 538 to 682 ms (*F* = 16.1, *p* = 0.001) and from 694 to 816 ms (*F* = 11.5, *p* = 0.004; see Figure 2C).

### 3.3 Analysis of ERFs to words preceded by function vs. content words, and close- vs. distinct-to-context words

We wanted to ensure that the late effects on the ERF differences we observed were not driven by the onset of the following word. To test this, we compared brain activity related to these following words separately for each condition (close-to-context vs. distinct-to-context; function vs. content words).

**Figure 3:**
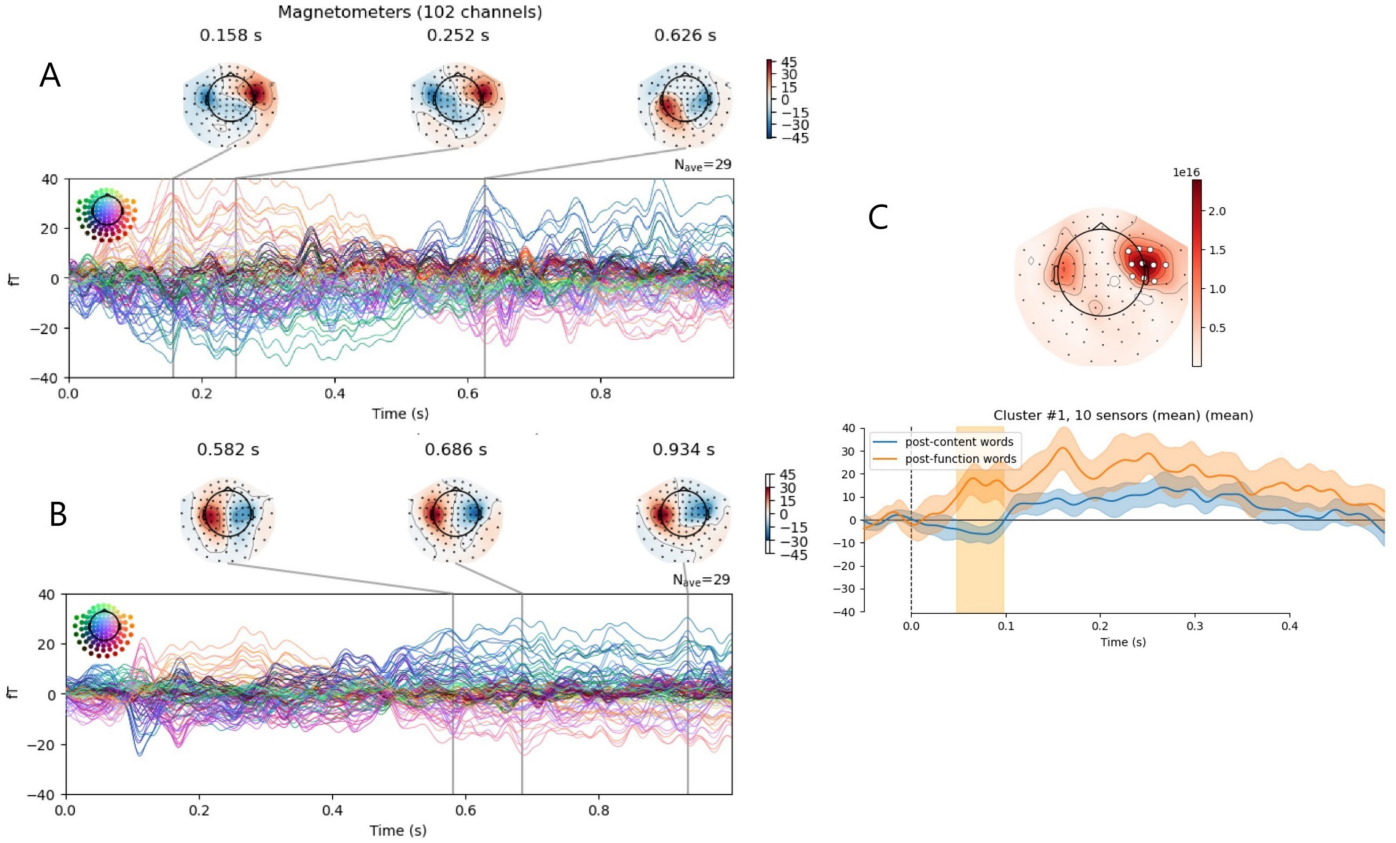
Event-related fields (ERFs) for words following function vs. content words. **(A,B)** ERFs shown for all channels in one timeline with highlighted peaks and their topography: **(A)** post-function words **(B)** post-content words. **(C)** comparison of ERFs to post-function and post-content words: Top panel—topography map with a significant sensor cluster marked in white, bottom panel—ERF averaged across this cluster; significant time periods are highlighted with yellow color. Confidence interval across participant data is shown.

At first, we compared the words following function words with those evoked by words following content words. Chi-square analysis showed a statistically significant difference in the proportion of function, content, close-to-context, and distinct-to-context words between the compared groups (χ^2^ = 34.6, *p* < 0.001), since very few function words followed function words. After removing function words from the compared groups (words that followed function and content words), Chi-square analysis showed no statistically significant difference in the proportion of content, close-to-context, and distinct-to-context words between the compared groups (χ^2^ = 0.62, *p* > 0.05). Permutational cluster analysis revealed one significant cluster in ERFs. At the right temporal sensors, words following function words induced more pronounced positive ERF in the time window from 48 to 98 ms (*F* = 15.3, *p* = 0.002; Figure 3C).

We also compared the ERFs evoked by words following close-to-context words with those evoked by words following distinct-to-context words. Chi-square analysis showed that there was no statistically significant difference in the proportion of function, content, close-to-context, and distinct-to-context words between the compared groups (χ2 = 0.97, p > 0.05). Permutational cluster analysis revealed no significant clusters (all *p*-values > 0.04, with cluster significance level = 0.005).

## 4 Discussion

In this study, we used MEG to investigate how semantic dissimilarity affects the neural processing of words in natural speech. We introduced an ERF-based approach for narrative auditory speech processing, enabling precise examination of the temporal dynamics of word context integration. We showed that distinct-to-context words elicited stronger late ERF responses at the left temporal sensors, suggesting increased processing demands. Additionally, we compared content and function words to explore how word type modulates ERFs during continuous auditory speech comprehension. Function words, characterized by their syntactic and predictive roles, elicited stronger early and late ERF responses, that might correspond to the next word tuning. Our results provide new evidence on how semantic dissimilarity and word class modulate neural responses during the comprehension of continuous natural speech. Below we discuss these findings in more detail.

### 4.1 Event-related fields (ERFs) for close-to-context and distinct-to-context words

Сlose- and distinct-to-context content words displayed different ERF profiles. While close-to-context words’ ERFs has pronounced early peaks that were rather typical for MEG auditory studies (Kaan, 2007; Kolozsvári, 2021; Salmelin, 2007; N'Diaye et al., 2004; Service et al., 2007), distinct-to-context words elicited mainly the late components (Figures 1A,B). At the same time - early differences between these ERFs were non-significant. The significant difference observed between ERFs to close- and distinct-to-context words in the 616–666 ms window at the left temporal sensors (Figure 1C). Here and throughout the Discussion, spatial references refer to sensor-level effects; no source reconstruction was performed, and anatomical localization is therefore not implied.

This window appears later than that reported by Broderick et al. (2018) in their TRF study of English (380–600 ms). Brodbeck et al. (2023) show that ERP may yield stronger responses during later intervals, which they attribute to acoustic leakage and overlap with subsequent words. However, our control analysis of subsequent words revealed no significant effects, which argues against overlap from following stimuli. We also controlled for word duration by excluding words shorter than 500 ms, confirming that in this case the late ERF differences between close- and distinct-to-context words persisted independently of word length (Fig. S1). Recently, a TRF analysis of semantic dissimilarity in natural Russian speech based on MEG data revealed a semantic-related response in the 200–400 ms window (Ovakimian et al., 2025), which is, on the contrary, earlier than the effect reported in the described study on English. The authors used a time window up to 600 ms after, which likely missed later responses. In the present study, longer epochs were analyzed. The observed late difference between high- and low-semantic-dissimilarity word may reflect ERP sensitivity to later processing stages. Brodbeck et al. (2023) also report that TRF tends to detect earlier effects, which may account for the earlier semantic-dissimilarity latency in Broderick et al. (2018) and in Ovakimian et al. (2025). Taken together, our results and their comparison with the literature suggest that combining ERP and TRF analyses can provide a more accurate estimate of the effect’s timing. While methodological differences may contribute to divergent latencies, linguistic factors should also be considered: Russian words are typically longer and morphologically more complex (with suffixes and case endings), whereas word order is flexible, which complicates syntactic parsing and may delay semantic integration.

Given the late timing and the known involvement of late potentials in working memory load during sentence processing, the observed component might correspond to the slow negative wave of ERP (Kaan, 2007; N'Diaye et al., 2004). However, it can also be suggested that the discovered difference is associated with the classic P600 component. Although the P600 wave is typically observed over central-parietal EEG electrodes, Service et al. (2007) using MEG suggested that its generators are located bilaterally in the temporal lobe. This component may reflect higher-order semantic processing of content words, specifically their integration into a coherent linguistic context. According to the literature, the P600 may arise during the processing of grammatically correct but semantically unexpected or complex words, when the integration of a word into context is difficult (Gunter et al., 2000; Service et al., 2007; Kaan, 2007). The P600 occurs in tasks that require judging the correctness of a word (Larionova and Martynova, 2022). Presumably, the P600 reflects a late stage of re-checking possible errors in the word, allowing distinguishing pseudowords, or errors in the word processing (Larionova and Garakh, 2024). The P600 has also been observed in tasks involving the establishment of new discourse referents, as well as violations of musical and mathematical structures (Kaan, 2007). This finding suggests the P600 reflects difficulty integrating stimuli into a preceding context, whether linguistic or not (Patel et al., 1998; Núñez-Peña and Honrubia-Serrano, 2004; Lelekov et al., 2000). In line with these findings, in our study, words with high semantic dissimilarity were more difficult to integrate into the unfolding sentence context, likely triggering such late verification processes, which may account for the observed P600-like enhancement. Importantly, semantic dissimilarity in natural speech is tightly coupled with lexical frequency and contextual predictability; thus, the increased integration cost observed here may reflect not only semantic distance itself, but also co-varying effects of lower frequency and higher surprisal, which were not explicitly controlled. Morphological complexity may have further contributed to integration difficulty, although its impact was partially reduced by excluding long words, given the correlation between word length and morphological complexity in Russian. It should also be noted that the observed late semantic effect should be referred to as “P600-like” with caution, as the present data do not provide sufficient evidence for a firm mechanistic interpretation.

To summarize, we observed a late ERF enhancement following semantically distant words. According to the Retrieval–Integration theory (Brouwer et al., 2012; Venhuizen and Brouwer, 2025), such late activity is thought to reflect the difficulty of integrating a word into the evolving sentence meaning. In Russian, flexible word order weakens linear cues about syntactic roles, and rich morphology increases the processing demands for establishing syntactic and semantic relations. These factors may contribute to greater integration effort, potentially explaining the enhanced late ERF response observed in our data.

### 4.2 Event-related fields (ERFs) for function and content words

According to the permutation cluster analysis, function words elicited a significantly greater ERF around 200–300 ms after word onset at the left temporo-parietal sensors (Figure 2C). Previous studies have shown that semantic processing can occur within 200 ms of word presentation (Hauk et al., 2006; MacGregor et al., 2012; Sulpizio et al., 2007; Sysoeva et al., 2007; Helenius et al., 2002). Additionally, some studies indicate that anticipatory mechanisms are involved in earlier processing stages, with N400-like effects appearing as early as in N200 responses (Brothers et al., 2015; Dikker and Pylkkanen, 2011). From about 200 ms onwards, the superior temporal activation shows sensitivity to lexical-semantic manipulation (Helenius et al., 2002; Kujala et al., 2004; Marinkovic et al., 2003). Although function words do not carry the main semantic load, they are critical for the syntactic structure of the sentence and have very short duration, which may require more active processing at early stages.

The permutation cluster analysis revealed stronger ERF for function words than for content words at late (450–650 ms) processing stages at anterior temporal sensors (Figure 2C). Previous studies have shown that function words can influence the N400 and have interpreted this effect as an indicator of anticipation of subsequent words (DeLong et al., 2005; Martin et al., 2008). The N400 has multiple interpretations, one of which is predictive coding theory, which posits that anticipated sensory inputs result in diminished neural responses (Eddine et al., 2024; Rabovsky and McRae, 2014). Thus, this observed later increase in ERF may reflect the role of function words in building expectations about the upcoming word and its expected syntactic structure, such as its grammatical case or grammatical features. As words unfold in spoken language, initial cues can trigger predictive processing, activating relevant memory traces and enabling the brain to anticipate an upcoming word well before the word is fully completed or recognized (Shtyrov and MacGregor, 2016).

Given the difference in word duration—function words averaging 167 ms and content words 394 ms—it is important to consider the possibility that the described late effect may be related to subsequent words. Therefore, we analyzed the activity associated with words following function and content words. According to this analysis, words that come after function words elicit a stronger brain response in the 50–100 ms range, particularly at the right anterior temporal sensors (Figure 3C). Given the same topography to the late ERF effect in response to function word, it can be suggested that this effect is likely related not to the processing of the function word itself, but rather to the predictive ability to process the following word. Importantly, this late effect may also partly reflect neural responses time-locked to the acoustic onset of the subsequent word, rather than processing of the function word itself. Function-content contrasts can reflect mixed linguistic and acoustic differences. However, in Russian prosodic and boundary structures provide limited differentiation between words and are therefore unlikely to substantially contribute to the observed early and late ERF effects. At the same time, function words also differ systematically from content words in duration, which may contribute to the enhanced early ERF response, independent of semantic processing.

Finally, cluster analysis revealed a greater activation for content words at the left sensors around the 550–800 ms range (Figure 2C). This difference seems to be linked to a late ERF wave that occurred for content words (Figure 2C), specifically, for distinct-to-context content words (Figures 1B,C), and was interpreted as a P600-like ERF reflecting the late semantic integration processing described in the previous section.

### 4.3 General conclusions

Overall, in the natural setting of listening to a story, close- and distinct-to-context content words elicited distinct ERF profiles, with distinct-to-context words showing a late (~600 ms) ERF component. This effect likely reflects difficulties in integrating them into the context, arising from semantic distance, reduced contextual predictability, or lower lexical frequency. Content words, which carry the primary semantic information, seem to trigger distinct late effects in ERFs at left temporal sensors, possibly linked to complex semantic integration and memory processes. On the other hand, stronger early ERF response at left parieto-temporal sensors to function words may reflect their role in syntactic processing, while the late ERF wave at right temporal sensors in the late stages could potentially indicate their involvement in predicting and processing the following words. Importantly, ERFs—when controlling for word duration and the influence of subsequent words—provide complementary information to TRF analyses and can help achieve a more complete understanding of semantic processing in natural speech.

## 5 Limitations

One limitation of natural auditory speech is the close temporal spacing between words, which complicates separating word-related activity from offset-related effects. However, the late effect around 600 ms showed stable latency across words of different durations, including analyses restricted to words shorter than 500 ms, arguing against an offset-based explanation. The early effect observed for function words (200–300 ms) is also unlikely to reflect offset-related activity, as no corresponding effect was found for content words at the expected latency. Despite controls for word duration and subsequent-word effects, temporal overlap and acoustic variability inherent to continuous speech cannot be fully excluded and may still influence neural responses to both content and function words, particularly given the short duration of function words. Вaseline contamination is a general concern in continuous-speech ERF analyses. However, the early effect extended beyond word offset, making a simple carry-over account unlikely.

Finally, the smaller number of function word trials may increase variability, but the effects remained robust across control analyses.

All results were obtained from a single speaker and a particular narrated story, which limits the generalizability of the findings to other speech materials and speaker-specific acoustic and phonetic properties.

## Data Availability

The datasets presented in this study can be found in online repositories. The names of the repository/repositories and accession number(s) can be found at: https://osf.io/4k7pe/.
