# Supplementary material for: Semantics and syntax effects on event-related fields during speech comprehension: a MEG study
Source: Front Hum Neurosci. 2026 Apr 2;20:1759177. doi: 10.3389/fnhum.2026.1759177 (PMC13083212; doi:10.3389/fnhum.2026.1759177)
Supplement: Supplementary file 1 [file Data_Sheet_1.pdf]

Supplementary Materials

1 Short words post-hoc analysis

More distinct-to-context than close-to-context words lasted beyond 500–600 ms (Fig. S1). To account for potential confounds related to ongoing word perception, we excluded all words longer than 500 ms from the analysis and examined whether the ERF difference between close- and distinct-to-context words could be attributed to this duration difference. As a post-hoc analysis, to neutralize the effect of word duration on the evoked potential latency, words with duration < 500 ms were selected from close- and distinct-to-context words. We used this duration threshold to determine whether the observed late effects (>500 ms) could be explained by the long duration (>500 ms) of distinct-to-context words. As a result, there were 112 short ‘close-to-context’ and 83 short ‘distinct-to-context’ words. We chose the 500 ms threshold because it yielded the most balanced and maximal number of words in the compared groups. The duration equality of the compared groups was additionally tested with unpaired t-test.

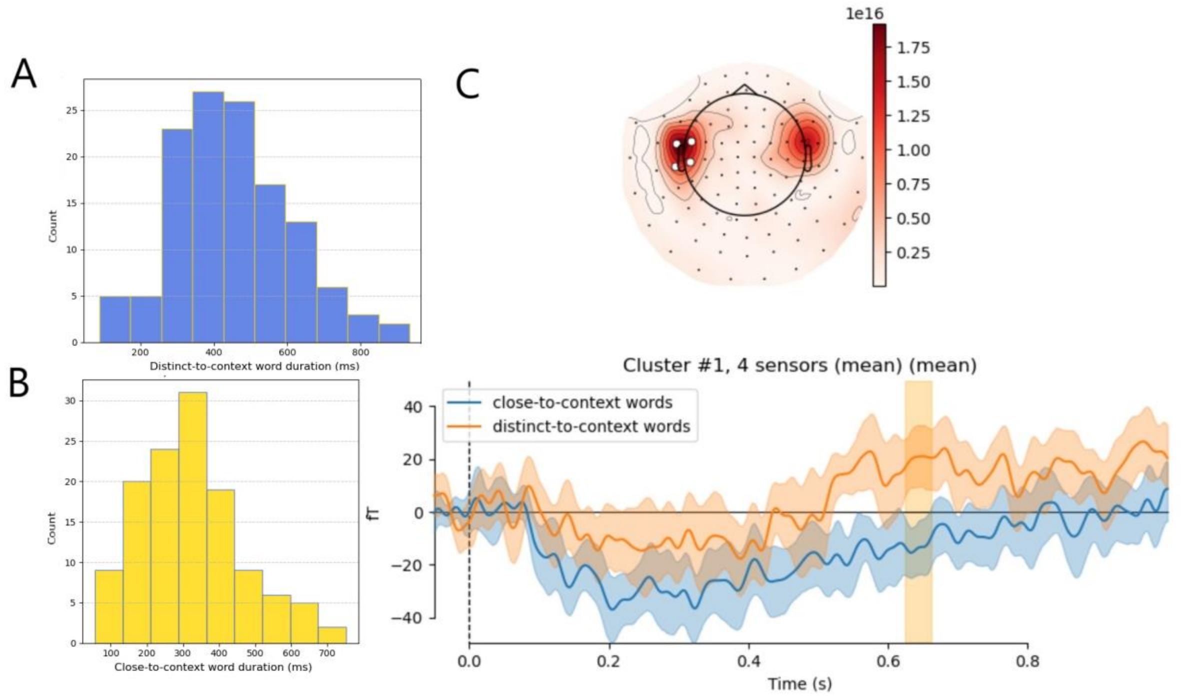

Figure S1 – Event-related fields (ERFs) for short (duration < 500 ms) close- and distinct-to-context words. A, B: Histograms of the duration distribution for all (A) distinct-to-context and (B) close-to-context words. C: Comparison of ERFs to close- and distinct-to-context short (<500 ms) words showing topography map with a significant sensor cluster marked in white and ERF averaged across this

cluster; significant time periods are highlighted with yellow color. Confidence interval across participant data is shown.

For this analysis, 112 short close-to-context words (for ex., 'live', 'hedgehogs', 'forests', 'animals', 'they') with duration shorter than 500 ms with mean = 286 ms (±106 ms) were selected. 83 short distinct-to-context words (for ex., 'vision', 'freedom', 'moisture') with duration shorter than 500 ms with mean = 359 ms (±96 ms) were selected.

Permutational cluster analysis revealed one significant cluster (Fig. S1). In left temporal sensors, distinct-to-context words induced more positive ERF than close-to-context words from 624 to 662 ms from word onset at the trend level ($F = 14.8$, $p = 0.03$ (with cluster significance threshold = 0.005)) that fully corresponds to the cluster revealed by the unrestricted to the word duration contrast.

2 Early ERF components

A visual inspection of the ERFs across individual channels revealed that, although the maximum amplitude peak of the ERFs and statistical differences are observed on the temporal channels, the early components of the ERFs appear slightly shifted, occurring closer to the left lateral parietal cortex (Fig. S2).

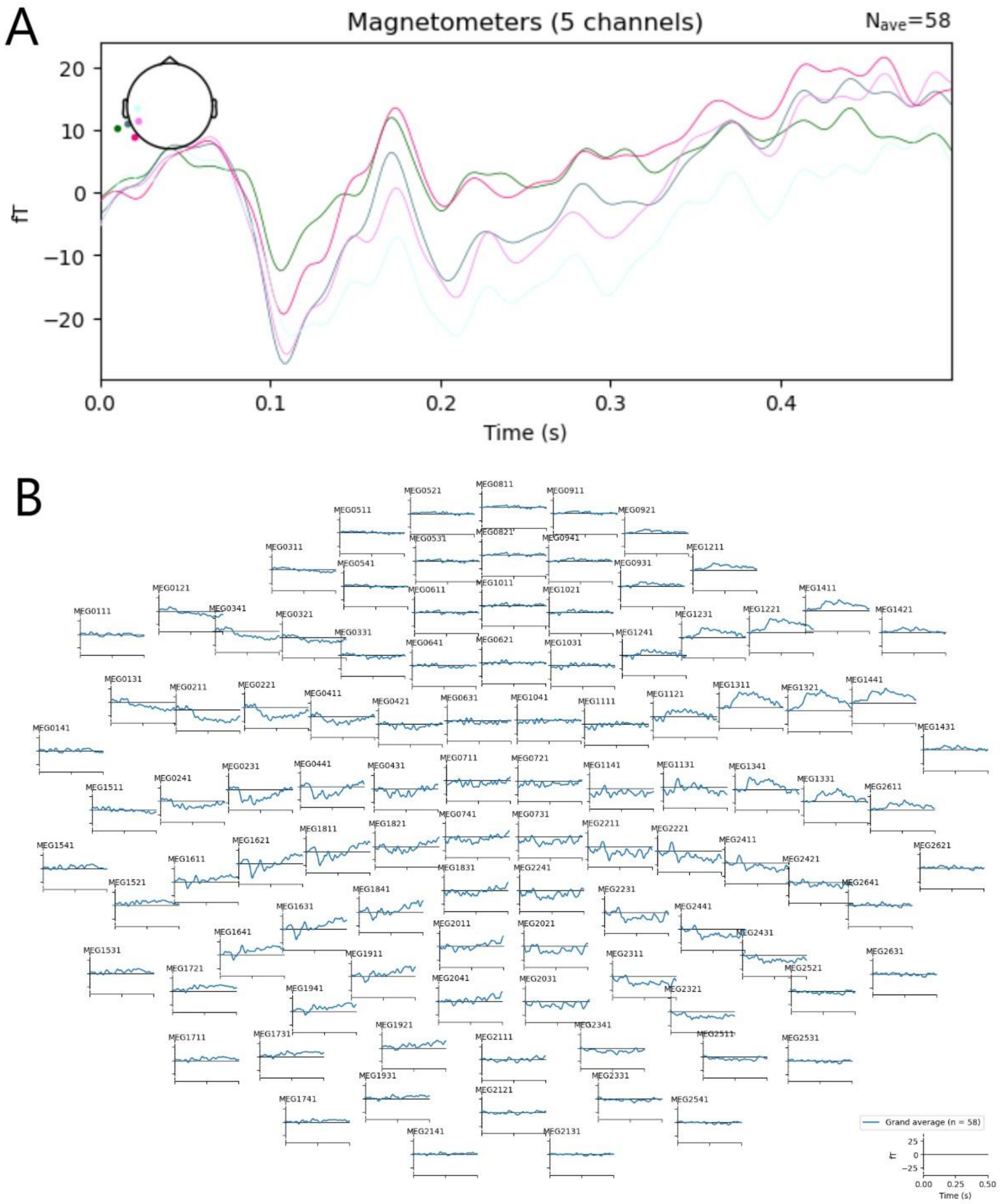

Figure 2 – Event-related fields (ERFs) for averaged close- and distinct-to-context words. A - Left lateral temporo-parietal channels, selected for visualization purposes, showing the early components of the ERF. B - Topography of ERFs.

While the peak ERF amplitude and the late statistical difference were observed at temporal sensors (Fig. 1), the early classic components of the auditory ERF (resembling N100, P100, N200, P200 and even N400; Kaan, 2007) were more pronounced in the temporal-parietal sensors in the left hemisphere. Speculatively,

different areas are involved in early and late auditory processing. It can be hypothesized that the cluster analysis for these short words revealed a more left anterior distribution compared to earlier components (similarly to left anterior negativity (Caffarra et al., 2019)), suggesting a shift to more frontal cortical sources for late processing, although further source localization is needed to confirm this hypothesis.

3 Text

Ежи которые живут в России называются обыкновенными. Их родственники рассеяны по всему свету и живут в лесах но только светлых где много густой травы и никаких болот на опушках в человеческих садах рядом с фермой и в парках. А вот белобрюхие ежи живут в Африке но только там где растут леса и есть влага. Китайские ежи живут в степи и ходят охотиться днем а не ночью как обыкновенные. Когда ёж рождается он очень маленький голый розовый и совсем без иголок. В длину ёжик с ладошку младенца а весит как несколько желудей. Ежата питаются маминым молоком и греются её теплом. Спустя две недели после рождения у ежат открываются глазки они начинают видеть. У них растут и крепнут иголки И так день за днём маленький ежик вырастает в большого ежа. Летом ежи спят совсем недолго всего три четыре часа потому что на улице тепло. А вот когда наступит зима они уснут на четыре месяца и будут спать всё время пока на земле лежит снег. У ежей на мордочке и животе есть шерсть а сверху на спине иголки. Иголки защищают ежей от врага и опасностей. Внутри иголки пустые но всё равно очень крепкие. Если ёж свернётся в клубок даже падать с высоты ему будет не страшно его убережёт жёсткий игольчатый шар. Иголки помогают ежам переносить полезные для жизни вещи. Только не красные круглые яблоки и грибы как на картинках а мох траву которые нужны ежам когда они строят норку. У ежей всего 35 зубов верхние очень острые чтобы откусывать а нижние потолще чтобы жевать. Ещё у ежей есть хвостик он совсем незаметный и маленький. Когти у ежей твёрдые и прочные их как и у людей пять по одному на каждый палец. А ещё ежи умеют топать своими лапами. Ежи способны очень быстро бегать. За секунду они могут пробежать три метра это очень быстро. Поэтому если враг ежа рядом то он не будет сворачиваться в клубок а убежит от него. Догнать ежа очень сложно. Кроме того у ежей хорошее обоняние и тонкий слух поэтому они чувствуют приближение врага издалека. Врагов у ежей много лисы волки хорьки и собаки. Но есть ещё и опасные птицы. Есть птицы которые просто поют и стучат клювом по дереву. Они не опасны. Но есть и те кто летают высоко глядят далеко и бросаются вниз как камни. Это филины. Они так же как и ежи просыпаются ночью и охотятся на всех ночных пешеходов. Спасенье

от филина только одно сжаться в комок. Ёж может съесть очень много за одну ночь он может съесть еды на треть от своего веса. Ежи питаются различной едой червяками насекомыми орехами грибами и ягодами. Есть легенда что в давние предавние времена в Италии жил огромный ёж в пять раз больше нынешних. Иголок у него не было зато был длинный хвост узкая мордочка и маленькие ушки. Он был размером с ребёнка. Ещё оказалось что иголки у ёжиков это не самое главное. Есть ежи совсем без иголок. У них густой мех длинный хвост равный примерно половине длины тела. От врагов они защищаются запахом. Пахнут зверьки луком и чесноком.А ещё они очень милые. А ещё есть очень милые ежи они называются ушастыми. У них действительно большие уши почти в половину длины головы. Иногда люди заводят домашних ежей. Они не топают и не сильно пахнут не впадают в зимнюю спячку а колючки у них не слишком колючие а окрасов великое множество. Но выпускают их в лес нельзя иначе домашнему ежу будет очень плохо. Весной летом и осенью люди с вертолётов и самолётов разбрасывают над лесами кусочки вкусной еды со спрятанными внутри лекарствами это специально для ёжиков чтобы они не заразились опасными болезнями. Но и сами ежи умеют лечиться. Они катаются в кислых осенних яблоках чтобы их сок попал в кожу под иголками и выгнал клещей и блох. Иногда после такого лечения кусочки яблок застревают между иголками и люди думают что ежи носят яблоки в свои гнёзда. Но это не так. Ежи не умеют носить ничего тяжёлого. И многие люди считают что ежи такие милые как котята. И очень их любят потому что они еще и полезны тем что едят вредных жуков.

4 Text translated

Hedgehogs that live in Russia are called common. Their relatives are scattered all over the world and live in forests, but only light ones where there is a lot of thick grass and no swamps, on the edges, in human gardens near a farm and in parks. But white-bellied hedgehogs live in Africa, but only where forests grow and there is moisture. Chinese hedgehogs live in the steppe and go hunting during the day and not at night like ordinary ones. When a hedgehog is born, it is very small, naked, pink and completely without needles. A hedgehog is as long as a baby's palm and weighs as many acorns. Hedgehogs feed on their mother's milk and warm themselves with its warmth. Two weeks after birth, the hedgehogs' eyes open and begin to see. Their needles grow and get stronger. And so day after day, a small hedgehog grows into a large hedgehog. In the summer, hedgehogs sleep for a very short time, only three to four hours, because it is warm outside. But when winter comes, they will fall asleep for four months and will sleep all the time while there is

snow on the ground. Hedgehogs have fur on their faces and stomachs and needles on top of their backs. The needles protect hedgehogs from enemies and dangers. The needles are empty inside, but still very strong. If a hedgehog curls up into a ball, even if it falls from a height, it will not be afraid, a hard needle ball will protect it. The needles help hedgehogs carry things that are useful for life. Just not red round apples and mushrooms like in the pictures, but moss and grass that hedgehogs need when they build a burrow. Hedgehogs have only 35 teeth, the upper ones are very sharp for biting off and the lower ones are thicker for chewing. Hedgehogs also have a tail, it is completely invisible and small. Hedgehogs have hard and strong claws, like humans, there are five of them, one for each finger. Hedgehogs can also stomp their paws. Hedgehogs can run very quickly. In a second they can run three meters, this is very fast. Therefore, if the hedgehog's enemy is nearby, it will not curl up into a ball, but will run away from it. It is very difficult to catch up with a hedgehog. In addition, hedgehogs have a good sense of smell and keen hearing, so they sense the approach of an enemy from afar. Hedgehogs have many enemies: foxes, wolves, ferrets and dogs. But there are also dangerous birds. There are birds that simply sing and knock their beaks on a tree. They are not dangerous. But there are those that fly high, look far away and throw themselves down like stones. These are eagle owls. They, like hedgehogs, wake up at night and hunt all night pedestrians. The only salvation from an eagle owl is to curl up into a ball. A hedgehog can eat a lot in one night, it can eat a third of its weight in food. Hedgehogs eat a variety of foods: worms, insects, nuts, mushrooms and berries. There is a legend that in ancient times in Italy there lived a huge hedgehog five times larger than the current ones. It had no needles, but it had a long tail, a narrow muzzle and small ears. It was the size of a child. It also turned out that needles are not the most important thing for hedgehogs. There are hedgehogs without needles at all. They have thick fur and a long tail equal to about half the length of their body. They protect themselves from enemies by smell. The animals smell like onions and garlic. And they are also very cute. And there are also very cute hedgehogs, they are called long-eared. They really have big ears, almost half the length of their head. Sometimes people get pet hedgehogs. They do not stomp and do not smell strongly, do not hibernate, and their spines are not too prickly, and they come in a great variety of colors. But they cannot be released into the forest, otherwise the domestic hedgehog will be very ill. In spring, summer and autumn, people from helicopters and planes scatter pieces of tasty food with medicines hidden inside over the forests, this is especially for hedgehogs so that they do not get infected with dangerous diseases. But hedgehogs themselves know how to heal themselves. They roll in sour autumn apples so that their juice gets into the skin under the needles and drives out ticks and fleas. Sometimes after such treatment, pieces of apples get stuck between the needles and people think that hedgehogs carry

apples to their nests. But this is not true. Hedgehogs do not know how to carry anything heavy. And many people think that hedgehogs are as cute as kittens. And they are very loved because they are also useful in that they eat harmful bugs.

5 Short (duration < 500 ms) close-to-context words:

'живут', 'ежи', 'живут', 'там', 'где', 'леса', 'есть', 'ежи', 'живут', 'как', 'совсем', 'ёжик', 'как', 'ежата', 'ежат', 'они', 'них', 'растут', 'так', 'маленький', 'ежик', 'ежи', 'совсем', 'потому', 'они', 'спать', 'всё', 'лежит', 'есть', 'ежей', 'всё', 'равно', 'ёж', 'ежам', 'грибы', 'как', 'мох', 'траву', 'ежам', 'ежей', 'потолще', 'ещё', 'ежей', 'хвостик', 'маленький', 'ежей', 'как', 'ещё', 'ежи', 'это', 'ежа', 'он', 'него', 'ежа', 'ежей', 'ежей', 'хорьки', 'ещё', 'как', 'это', 'так', 'как', 'ежи', 'филина', 'ёж', 'он', 'ежи', 'грибами', 'ёж', 'него', 'зато', 'он', 'ещё', 'это', 'есть', 'ежи', 'зверьки', 'ещё', 'милые', 'есть', 'ежи', 'они', 'них', 'ежей', 'они', 'них', 'их', 'лес', 'ежу', ‘лесами', 'это', 'они', 'сами', 'ежи', 'они', 'их', 'иногда', 'такого', 'ежи', 'это', 'так', 'ежи', 'ежи', 'такие', 'милые', 'как', 'котята', 'их', 'потому', 'они', 'еще', 'едят'.

6 Short close-to-context words translated:

'live', 'hedgehogs', 'live', 'there', 'where', 'forests', 'there is', 'hedgehogs', 'live', 'how', 'completely', 'hedgehog', 'how', 'hedgehogs', 'hedgehogs', 'they', 'they', 'grow', 'so', 'small', 'hedgehog', 'hedgehogs', 'completely', 'because', 'they', 'sleep', 'all', 'lies', 'there is', 'hedgehogs', 'all', 'anyway', 'hedgehog', 'hedgehogs', 'mushrooms', 'how', 'moss', 'grass', 'hedgehogs', 'hedgehogs', 'thicker', 'more', 'hedgehogs', 'tail', 'small', 'hedgehogs', 'how', 'more', 'hedgehogs', 'this', 'hedgehog', 'he', 'him', 'hedgehog', 'hedgehogs', 'hedgehogs', 'ferrets', 'more', 'how', 'this', 'so', 'how', 'hedgehogs', 'owl', 'hedgehog', 'he', 'hedgehogs', 'mushrooms', 'hedgehog', 'him', 'but', 'he', 'more', 'this', 'there is', 'hedgehogs', 'little animals', 'more', 'cute', 'there is', 'hedgehogs', 'they', 'them', 'hedgehogs', 'they', 'them', 'their', 'forest', 'hedgehog', 'forests', 'this', 'they', 'themselves', 'hedgehogs', 'they', 'their', 'sometimes', 'such', 'hedgehogs', 'this', 'so', 'hedgehogs', 'hedgehogs', 'such', 'cute', 'how', 'kittens', 'their', 'because', 'they', 'still', 'eat'.

7 Short (duration < 500 ms) distinct-to-context words:

'ежи', 'которые', 'живут', 'россии', 'всему', 'фермой', 'парках', 'влага', 'днем', 'ёж', 'он', 'очень', 'иголок', 'длину', 'ладошку', 'весит', 'её', 'теплом', 'спустя', 'две', 'крепнут', 'часа', 'улице', 'тепло', 'шерсть', 'сверху', 'спине', 'высоты', 'переносить', 'полезные', 'жизни', 'вещи', 'нужны', 'строят', тридцать, 'нижние', 'пять', 'умеют', 'топать', 'способны', 'секунду', 'могут', 'пробежать', 'три', 'рядом', 'догнать', 'обоняние', 'слух', 'приближение', 'издалека', 'поют', 'стучат',

'глядят', 'вниз', 'ночных', 'сжаться', 'может', 'ночь', 'может', 'еды', 'треть', 'веса', 'времена', 'ушки', 'равный', 'длины', 'тела', 'длины', 'заводят', 'топают', 'впадают', 'окрасов', 'великое', 'над', 'попал', 'кожу', 'выгнал', 'между', 'носят', 'носить', 'любят', 'полезны', 'жуков'.

8 Short distinct-to-context words translated:

'hedgehogs', 'which', 'live', 'russia', 'everything', 'farm', 'parks', 'moisture', 'day', 'hedgehog', 'he', 'very', 'needles', 'length', 'palm', 'weighs', 'her', 'warmth', 'after', 'two', 'get stronger', 'hours', 'street', 'warmth', 'wool', 'from above', 'back', 'heights', 'carry', 'useful', 'life', 'things', 'needed', 'build', thirty, 'lower', 'five', 'can', 'stomp', 'capable', 'second', 'can', 'run', 'three', 'near', 'catch up', 'smell', 'hearing', 'approaching', 'from afar', 'sing', 'knock', 'look', 'down', 'night', 'shrink', 'may', 'night', 'may', 'food', 'third', 'weight', 'times', 'ears', 'equal', 'lengths', 'bodies', 'lengths', 'start', 'stomp', 'fall', 'colors', 'great', 'over', 'got', 'skin', 'kicked out', 'between', 'wear', 'to wear', 'love', 'useful', 'beetles'.

9 Function words

'в', 'по', 'и', 'в', 'но', 'только', 'и', 'на', 'в', 'с', 'и', 'в', 'а', 'вот', 'в', 'но', 'только', 'и', 'в', 'и', 'а', 'не', 'когда', 'и', 'без', 'в', 'с', 'а', 'и', 'после', 'у', 'у', 'и', 'И', 'за', 'в', 'что', 'на', 'а', 'вот', 'когда', 'на', 'и', 'на', 'у', 'на', 'и', 'а', 'на', 'от', 'и', 'но', 'если', 'в', 'даже', 'с', 'не', 'для', 'только', 'не', 'и', 'на', 'а', 'когда', 'у', 'чтобы', 'а', 'чтобы', 'у', 'и', 'у', 'и', 'и', 'у', 'по', 'на', 'а', 'за', 'если', 'не', 'в', 'а', 'от', 'кроме', 'у', 'и', 'у', 'и', 'но', 'и', 'просто', 'и', 'по', 'не', 'но', 'и', 'и', 'же', 'и', 'и', 'на', 'от', 'только', 'в', 'за', 'на', 'от', 'и', 'что', 'в', 'в', 'в', 'у', 'не', 'и', 'с', 'что', 'у', 'не', 'без', 'у', 'от', 'и', 'а', 'а', 'у', 'в', 'не', 'и', 'не', 'не', 'в', 'а', 'у', 'не', 'а', 'но', 'в', 'и', 'с', 'и', 'со', 'для', 'чтобы', 'не', 'но', 'и', 'в', 'чтобы', 'в', 'под', 'и', 'и', 'после', 'и', 'что', 'в', 'но', 'не', 'не', 'и', 'что', 'и', 'что', 'и', 'что'
